# PT-Symmetric Potentials from the Confluent Heun Equation

**DOI:** 10.3390/e23010068

**Published:** 2021-01-03

**Authors:** Géza Lévai

**Affiliations:** Institute for Nuclear Research (Atomki), P. O. Box 51, H-4001 Debrecen, Hungary; levai@atomki.hu

**Keywords:** confluent Heun differential equation, solvable potentials, PT symmetry, 03.65.Ge, 02.30.Gp, 11.30.Er

## Abstract

We derive exactly solvable potentials from the formal solutions of the confluent Heun equation and determine conditions under which the potentials possess PT symmetry. We point out that for the implementation of PT symmetry, the symmetrical canonical form of the Heun equation is more suitable than its non-symmetrical canonical form. The potentials identified in this construction depend on twelve parameters, of which three contribute to scaling and shifting the energy and the coordinate. Five parameters control the z(x) function that detemines the variable transformation taking the Heun equation into the one-dimensional Schrödinger equation, while four parameters play the role of the coupling coefficients of four independently tunable potential terms. The potentials obtained this way contain Natanzon-class potentials as special cases. Comparison with the results of an earlier study based on potentials obtained from the non-symmetrical canonical form of the confluent Heun equation is also presented. While the explicit general solutions of the confluent Heun equation are not available, the results are instructive in identifying which potentials can be obtained from this equation and under which conditions they exhibit PT symmetry, either unbroken or broken.

## 1. Introduction

The efforts of extending and perfecting the mathematical formulation of quantum mechanics are as old as quantum mechanics itself. A remarkable new direction of these studies was started in 1998 with the introduction of PT-symmetric quantum mechanics [1]. In this construction the traditional hermiticity requirement of the Hamiltonian is replaced by a symmetry property prescribing invariance with respect to the simultaneous space (P) and time (T) inversion. In the case of one-dimensional non-relativistic quantum mechanical potential problems this requirement leads to the Schrödinger equation with potentials satisfying $V^{*}\left(-x\right)=V\left(x\right)$. Surprisingly, these complex potentials were found to exhibit properties similar to those of conventional (real) potentials.

It turned out that the discrete energy spectrum of many PT-symmetric potentials contains fully or partly real energy eigenvalues. Furthermore, it was also found that by tuning the potential parameters (typically by increasing non-hermiticity), real energy eigenvalues merged pairwise and re-emerged as complex conjugate pairs. Since in this process the PT symmetry of the Hamiltonian remained intact, while the wave functions ceased to be the eigenfunctions of the PT operator, this phenomenon was interpreted as the breakdown of PT symmetry. It also turned out that the energy spectrum of some potentials always remains real, irrespective of the choice of the potential parameters.

These first results naturally triggered studies to clarify under which conditions the complexification of the energy eigenvalues can occur. Besides their energy spectrum, PT-symmetric potentials exhibited further unusual properties. The conventional hermitian inner product had to be replaced with a PT inner product, which, however, led to indefinite norm [2]. Studies were started to investigate the mapping between PT-symmetric quantum systems and their hermitian correspondents. It also turned out that non-hermitian constructions have already been applied before in various branches of quantum physics, and PT-symmetric quantum mechanics offered a theoretical scheme to discuss them in a unified framework. PT-symmetric quantum mechanics was identified as a special case of pseudo-hermiticity [3,4,5]. The results and ideas of the new theory found applications in various branches of physics to discuss as diverse topics as e.g., neutrino oscillations [6] and conformal gravity [7]. In optics, the existence of PT-symmetric systems in nature, as well as the breakdown of PT symmetry was verified experimentally [8]. See reference [9] for a recent comprehensive review of PT-symmetric quantum mechanics and [10] for a more mathematically oriented discussion of the subject. PT-symmetric quantum mechanics can also be related to theories developed before its introduction, e.g., optical potentials [11] and open quantum systems [12].

In exploring the main features and the capabilities of any new theory, exactly solvable models serve as invaluable tools. While the first examples for PT-symmetric quantum mechanics were numerical models, exactly solvable examples were soon presented too. It turned out that techniques developed for the exact solution of conventional quantum mechanical potential problems can be adapted to PT-symmetric setting too. The first analytically solvable examples belonged to the shape-invariant potential class [13], which contains the most well-known textbook examples for solvable potentials (harmonic oscillator, Coulomb, Pöschl–Teller, etc.). Exact results have been obtained for bound states with real [14] and complex [15] energy eigenvalues, the mechanism of the breakdown PT symmetry [16], the pseudo-norm of wavefunctions [17,18,19,20], the C operator [21], spectral singularities [22], supersymmetric [23,24], higher dimensional [25,26,27] and algebraic [28,29,30] aspects, etc. See Chapter 7 of reference [9] for a review of exactly solvable potentials and their adaptation to PT symmetry.

Later some potentials from the more general six-parameter Natanzon [31] class have also been discussed in the PT-symmetric setting. The general solutions of these potentials are written in terms of the hypergeometric function, which typically reduces to a Jacobi polynomial for bound states. (Shape-invariant potentials are special cases of the Natanzon class with 2 or 3 parameters.) The PT-symmetric versions of Natanzon-class potentials exhibited various character from the point of view of their energy spectrum: some had purely real energy eigenvalues [32], in some other the complexification of the energy eigenvalues occurred at the same parameter value for all the levels [33], similarly to some shape-invariant potentials, while there were also examples for which the complexification of the energy eigenvalues occurred gradually as the key potential parameter was tuned [34]. These results called for a systematic exploration of the PT-symmetrization of Natanzon-class potentials. This project was presented in reference [35], where all known Natanzon-class potentials have been classified and discussed in the PT-symmetric setting. An important finding of this study was that in the adaptation of PT symmetry to these potentials it is more favorable to set out from the differential equation of the Jacobi polynomials than starting from the hypergeometric differential equation.

There are, however, even wider potential classes with higher number of potential parameters. The solutions of these potential problems are written in terms of functions satisfying second-order differential equations with more general structure than the hypergeometric differential equation. Examples for this are various variants of the Heun differential equation [36]: Heun’s equation, the confluent, double confluent, biconfluent and triconfluent Heun equation. These equations differ from each other in the number and character of their singular points. Heun’s equation can also be reduced to the hypergeometric differential equation. However, a major difference is that in contrast with the hypergeometric differential equation, the solutions of which, the hypergeometric functions can be written in closed form, the solutions of the Heun (and related) equation are much less known. There are various methods to expand them in terms of known functions or power series. See e.g., references [37,38,39,40,41,42,43,44,45,46], for a sample of studies in which the solutions of the various Heun equations were expanded in terms of (confluent) hypergeometric and other functions.

Here we discuss the confluent Heun equation, which has regular singularities at two finite points (conventionally z=0 and 1), and an irregular singularity at z→∞. It can also be considered a generalization of the hypergeometric and the confluent hypergeometric differential equation, and this can open the way to the generalization of known Natanzon-class potentials. A comprehensive survey of potentials that can be discussed in terms of the confluent Heun differential equation has been presented in reference [47]. (See also the much earlier reference [48], where potentials derivable from the Heun, confluent, biconfluent and double confluent Heun equation are discussed in some detail.) The general structure of the potentials has been outlined, together with the solutions written formally in terms of confluent Heun functions. However, since these functions are not known in closed form in general, the complete solutions (with energy eigenvalues) could not be presented. Here we revisit this problem with the intention of implementing PT symmetry to potentials that can be derived from the confluent Heun differential equation. It turns out that a number of non-trivial conditions can be formulated for the construction of PT-symmetric potentials even without knowing the explicit form of the solutions.

The structure of the paper is as follows. In Section 2 a standard method of constructing solvable potentials is outlined. It is then applied to various forms of the confluent Heun equation in Section 3, and it is shown that its symmetrical canonical form is more suitable for the construction of PT-symmetric potentials. The actual implementation of PT symmetry is carried out in Section 4, where a survey of prospective PT-symmetric potentials and their solutions is presented. Finally, the results are summarised in Section 5.

## 2. Exactly Solvable Potentials from Special Functions of Mathematical Physics

Since the one-dimensional Schrödinger equation is a second-order differential equation, it is a natural choice to search for its solutions in terms of those special functions of mathematical physics that satisfy a second-order differential equation. In this case a simple variable transformation can be used to map the two differential equations into each other.

Let us consider the second-order differential equation of a special function F(z),

(1) $$\frac{d^{2}F}{dz^{2}}+Q\left(z\right)\frac{dF}{dz}+R\left(z\right)F\left(z\right)=0\;,$$

which we map into the Schrödinger equation

(2) $$\frac{d^{2}\psi }{dx^{2}}+\left(E-V\left(x\right)\right)\psi \left(x\right)=0\;,$$

by applying the variable transformation z(x). (For the sake of simplicity the units 2m=1 and ℏ=1 are used.) For this we substitute

(3) ψ(x)=f(x)F(z(x)),

into Equation (2). Comparing the appropriate terms, elementary calculation leads to the equation

(4) $$\begin{array}{ccc} E-V(x) & = & \frac{z^{\prime \prime \prime }\left(x\right)}{2z^{\prime }\left(x\right)}-\frac{3}{4}{\left(\frac{z^{\prime \prime }\left(x\right)}{z^{\prime }\left(x\right)}\right)}^{2}\\ & & +{\left(z^{\prime }\left(x\right)\right)}^{2}\left(R\left(z\left(x\right)\right)-\frac{1}{2}\frac{dQ}{dz}-\frac{1}{4}Q^{2}\left(z\left(x\right)\right)\right). \end{array}$$

Furthermore, one also finds that the solutions of the Schrödinger equation can be expressed in terms of the special function F(z) as

(5) $$\psi \left(x\right)∼{\left(z^{\prime }\left(x\right)\right)}^{-\frac{1}{2}}\exp\left(\frac{1}{2}\int^{z(x)}Q\left(z\right)dz\right)F\left(z\left(x\right)\right)\;.$$

Now the potential and the wave functions are given in terms of the functions Q(z) and R(z) that define the special function F(z), and the z(x) function that defines the variable transformation. Selecting the special function, the only remaining task is to find an appropriate z(x) function. Generally, any reasonable function (e.g., sigle-valued monotonous) would do, however, one cannot guarantee that the solutions would belong to the same potential V(x) and different *E* energy eigenvalues. A possible choice was proposed by Bhattacharjie and Sudarshan [49], who noticed that in order to reproduce a constant (*E*) on the left handside of Equation (4), there must be a constant on the right handside too, and this prescription defines a differential equation for z(x). Furthermore, it is reasonable to consider terms from those depending on Q(z) and R(z), because these terms contain the parameters that appear in the special function F(z). The first two terms of Equation (4) with higher derivatives of z(x) are called Schwartzian derivative, and contain only those parameters that define the variable transformation.

Considering the differential equation

(6) $${\left(\frac{dz}{dx}\right)}^{2}\Phi \left(z\right)=C\;,$$

its integration immediately leads to

(7) $$\int \Phi^{1/2}\left(z\right)dz=C^{1/2}x+x_{0}$$

defining the inverse x(z) function, which is not necessarily invertible. In the case of non-invertible x(z) the potential is called *implicit*, nevertheless, the procedure can be used to generate the solutions (5) and the potential V(x) even then. Here $x_{0}$ is a constant of integration that represents a coordinate shift that has no effect on the energy spectrum. It is usually unimponrtant and can be chosen such that z(0)=0. However, in the case of PT-symmetric quantum mechanics it can play an important role, as discussed later.

Combining Equations (4) and (6), $z^{\prime }\left(x\right)$ can be replaced in the former expression, leaving V(x) and *E* as the function of Q(z), R(z), Φ(z), z(x) and *C*:

(8) $$\begin{array}{ccc} E-V(x) & = & \frac{z^{\prime \prime \prime }\left(x\right)}{2z^{\prime }\left(x\right)}-\frac{3}{4}{\left(\frac{z^{\prime \prime }\left(x\right)}{z^{\prime }\left(x\right)}\right)}^{2}\\ & & +\frac{C}{\Phi (z(x))}\left(R\left(z\left(x\right)\right)-\frac{1}{2}\frac{dQ}{dz}-\frac{1}{4}Q^{2}\left(z\left(x\right)\right)\right). \end{array}$$

When F(z) is chosen to be the hypergeometric function ${}_{2}F_{1}\left(a,b;c;z\right)$, one obtains the Natanzon-class potentials [31], which depend on six parameters. Three of these appear in the z(x) function, while the remaining three arise from *a*, *b* and *c* and play the role of coupling coefficients in the potential V(x). It was found that for specific choices of the first three parameters one obtains the shape-invariant potentials [13], which are the most well-known textbook examples for exactly solvable potentials (Pöschl-Teller, Scarf, Rosen-Morse, Eckart). Three further shape-invariant potentials, the Morse, radial harmonic oscillator and Coulomb can be derived in a similar way as special cases of confluent Natanzon potentials [50], setting out from the confluent hypergeometric differential equation.

For a=−n or b=−n the hypergeometric function reduces to the Jacobi polynomial $P_{n}^{\left(\alpha ,\beta \right)}\left(y\right)$, where y=(1−z)/2 [51]. Applying the procedure directly to the Jacobi polynomials one finds that the shape-invariant potentials mentioned above are 1+2 parameter members of the Natanzon potential class in the sense that one parameter controls the variable transformation, while two others appear in the coupling coefficients of two independently tunable potential terms. In principle, the same potentials can be obtained setting out either from the hypergeometric function or the Jacobi polynomial. The equivalence of the two precedures has been demonstrated in reference [35]. However, in certain circumstances one of the procedures has advantages over the other one. As it was mentioned in the Introduction, the discussion of PT-symmetric potentials is more straightforward if one applies Jacobi polynomials. This is because when the function defining the variable transformation is PT-even or PT-odd, i.e., PTy(x)=±y(x) holds, then the construction of a PT-symmetric potential is much more straightforward. Note that z(x)=1−2y(x), which appears in the argument of the hypergeometric function, has no definite PT symmetry if y(x) is PT-odd.

## 3. Application to the Confluent Heun Equation

The non-symmetrical canonical form of the confluent Heun equation corresponds to Equation (1) with

(9) $$Q\left(z\right)=4p+\frac{\gamma }{z}+\frac{\delta }{z-1}\;,$$

(10) $$R\left(z\right)=\frac{4p\alpha z-\sigma }{z(z-1)}\;,$$

and F(z)=Hc(p,α,γ,δ,σ;z) is its solution [36]. Note that the hypergeometric differential equation is obtained for p=0, γ=c, δ=a+b+1−c and σ=−ab.

The potentials generated from this equation have been determined by Iskhanyan [47] using a method that is closely related to that outlined in Section 2. It was found that

(11) $$V\left(x\right)=-\frac{z^{\prime \prime \prime }\left(x\right)}{2z^{\prime }\left(x\right)}+\frac{3}{4}{\left(\frac{z^{\prime \prime }\left(x\right)}{z^{\prime }\left(x\right)}\right)}^{2}+\frac{v_{0}+v_{1}z+v_{2}z^{2}+v_{3}z^{3}+v_{4}z^{4}}{r_{0}+r_{1}z+r_{2}z^{2}+r_{3}z^{3}+r_{4}z^{4}}$$

and

(12) $$\frac{dz}{dx}=\pm z\left(z-1\right){\left(r_{0}+r_{1}z+r_{2}z^{2}+r_{3}z^{3}+r_{4}z^{4}\right)}^{-1/2}\;.$$

The same results can be reached using the procedure outlined in Section 2.

In reference [47] a different method was followed to generate solvable potentials. It was assumed that $z^{\prime }\left(x\right)$ is expressed in terms of the integer or half-integer powers of *z* and z−1, i.e.,

(13) $$\frac{dz}{dx}=\pm \sigma^{-1}z^{m_{1}}{\left(z-1\right)}^{m_{2}}\;,$$

where $m_{1}\leq 1$, $m_{2}\leq 1$ and $m_{1}+m_{2}\geq 0$. This corresponds to taking $C=\sigma^{2}$ and $\Phi \left(z\right)=z^{2-2m_{1}}{\left(z-1\right)}^{2-2m_{2}}$ in Equation (6). With this choice the terms originating from the Schwartzian derivative turn out to be a limited set of powers of 1/z and/or 1/(z−1), which allows their combination with the potential terms derived from Q(z) and R(z). Neglecting this specific choice, more general Φ(z) functions can be considered at the price that potential terms containing fixed coupling coefficients and powers ${\left(\Phi \left(z\right)\right)}^{-k}$, k=1,23 appear in V(x).

Following the analysis presented in reference [35] concerning the parity properties of z(x) and its derivatives, one finds that the parity of z(x), Φ(z(x)) and V(x) cannot be controlled in a simple way. For example, if z(x) is even or odd function of *x*, then z(x)−1 will be even in the first case, but will have indefinite parity in the latter. Determining the parity of Φ(z(x)), the potential and the wave functions will be even more complicated. Since the parity (P) property of these quantities plays an essential role in PT-symmetric quantum mechanics too, one finds that this approach is not ideal when it comes to constructing PT-symmetric potentials. As it was mentioned in the Introduction, the situation is analogous to that desribed in reference [35], where the Natanzon-class potentials were discussed from the point of view of PT symmetry. The general solutions of these potentials are given in terms of the hypergeometric function, which reduce to Jacobi polynomials for bound states. Instead of *z* and z−1 appearing in the expressions for hypergeometric functions, one can use 1−z and 1+z in the case of Jacobi polynomials. If z(x) has definite parity, then so will $\left(1-z\right)\left(1+z\right)=1-z^{2}$, while this is not the case for z(z−1).

These considerations indicate that implementing PT symmetry the symmetrical canonical form of the confluent Heun equation [36] will be more favorable. This choice corresponds to the following forms of Q(z) and R(z) in Equation (1):

(14) $$Q\left(z\right)=-2p-\frac{m+s+1}{1-z}+\frac{m-s+1}{1+z}$$

and

(15) $$R\left(z\right)=\frac{-2p(B-m-1)z-2ps-m(m+1)+\lambda }{\left(1-z)(1+z\right)}\;.$$

Note that the differential equation reduces to that of the Jacobi polynomials for

(16) p=0,m=(α+β)/2,s=(α−β)/2,λ=(n+(α+β)/2)(n+1+(α+β)/2).

With the choices (14) and (15) the current form of Equation (4) becomes

(17) $$\begin{array}{ccc} E-V(x) & = & \frac{z^{\prime \prime \prime }\left(x\right)}{2z^{\prime }\left(x\right)}-\frac{3}{4}{\left(\frac{z^{\prime \prime }\left(x\right)}{z^{\prime }\left(x\right)}\right)}^{2}+\frac{{\left(z^{\prime }\left(x\right)\right)}^{2}}{{\left(1-z^{2}\left(x\right)\right)}^{2}}\left[\lambda (1-z^{2}\left(x\right))-2msz\left(x\right)\right.\\ & & \left.+\left(1-m^{2}-s^{2}\right)-2pBz\left(x\right)\left(1-z^{2}\left(x\right)\right)-p^{2}{\left(1-z^{2}\left(x\right)\right)}^{2}\right], \end{array}$$

while Equation (6) turns into

(18) $${\left(\frac{dz}{dx}\right)}^{2}\Phi \left(z\left(x\right)\right)\equiv {\left(\frac{dz}{dx}\right)}^{2}\frac{\phi (z(x))}{{\left(1-z^{2}\left(x\right)\right)}^{2}}=C\;,$$

i.e.,

(19) $$\frac{dz}{dx}=\pm C^{1/2}\left(1-z^{2}\left(x\right)\right){\left[\phi \left(z\left(x\right)\right)\right]}^{-1/2}\;,$$

where

(20) $$\phi \left(z\left(x\right)\right)=p_{I}\left(1-z^{2}\left(x\right)\right)+p_{\mathrm{II}}+p_{\mathrm{III}}z\left(x\right)+p_{\mathrm{IV}}z\left(x\right)\left(1-z^{2}\left(x\right)\right)+p_{V}{\left(1-z^{2}\left(x\right)\right)}^{2}\;.$$

Since the solutions of the symmetrical canonical form of the Heun equation can be expressed in terms of the solutions of the non-symmetrical case by taking the substitutions [36]

(21) $$\begin{array}{c} \gamma =m+s+1,\;\delta =m-s+1,\;\alpha =-B+m+1,\\ \sigma =\lambda -m(m+1)-2p(B-m-s-1) \end{array}$$

and z→1−2z, the unnormalized solutions of the potentials obtained from the symmetrical canonical form of the Heun equation will, according to Equation (5) take the form

(22) $$\begin{array}{ccc} \psi (x) & ≃ & \phi^{\frac{1}{4}}\left(z\left(x\right)\right)\exp\left(-pz\left(x\right)\right){\left(1+z\left(x\right)\right)}^{\frac{1}{2}\left(m-s\right)}{\left(1-z\left(x\right)\right)}^{\frac{1}{2}\left(m+s\right)}\\ & & \times Hc(p,-B+m+1,m+s+1,m-s+1,\lambda -m\left(m+1\right)-2p\left(B-m-s-1\right);\frac{1}{2}\left(1-z\left(x\right)\right)\;. \end{array}$$

The x(z) function, and whenever possible, z(x) is then determined from the integration of Equation (19). The domain of definition of z(x) depends on the actual situation: it can be the full real *x* axis, a finite section of it, or the positive real axis. In the case of PT-symmetric problems the potentials may be defined on trajectories in the complex *x* plane too. In this case the coordinate shift $x_{0}$ can be chosen imaginary, in order to avoid singularities at x=0, for example. This mechanism is useful in constructing PT-symmetric problems from hermitian ones by extending them to negative real *x* values too [14].

From the structure of (17) one can conclude that the most general form of the derived potential is

(23) $$\begin{array}{ccc} V(x) & = & -\frac{z^{\prime \prime \prime }\left(x\right)}{2z^{\prime }\left(x\right)}+\frac{3}{4}{\left(\frac{z^{\prime \prime }\left(x\right)}{z^{\prime }\left(x\right)}\right)}^{2}+\frac{C}{\phi (z(x))}\left[s_{I}\left(1-z^{2}\left(x\right)\right)+s_{\mathrm{II}}\right.\\ & & \left.+s_{\mathrm{III}}z\left(x\right)+s_{\mathrm{IV}}z\left(x\right)\left(1-z^{2}\left(x\right)\right)+s_{V}{\left(1-z^{2}\left(x\right)\right)}^{2}\right]. \end{array}$$

Here, and in what follows we extend the notation used in reference [35] such that the results obtained for the special parameters (16) recover the formulas relevant to the Natanzon-class potentials with Jacobi polynomials in their bound-state solutions.

The functional form of V(x) can be made more explicit by substituting Equation (19) and its derivatives into the terms of the Schwartzian derivative. One finds that

(24) $$\begin{array}{ccc} -\frac{z^{\prime \prime \prime }\left(x\right)}{2z^{\prime }\left(x\right)}+\frac{3}{4}{\left(\frac{z^{\prime \prime }\left(x\right)}{z^{\prime }\left(x\right)}\right)}^{2} & = & \frac{C}{\phi (z(x))}-\frac{5}{16}\frac{C}{\phi^{3}\left(z\left(x\right)\right)}{\left(1-z^{2}\left(x\right)\right)}^{2}{\left(\frac{d\phi }{dz}\right)}^{2}\\ & & +\frac{C}{\phi^{2}\left(z\left(x\right)\right)}\left[-\frac{1}{2}z\left(x\right)\left(1-z^{2}\left(x\right)\right)\frac{d\phi }{dz}+\frac{1}{4}{\left(1-z^{2}\left(x\right)\right)}^{2}\frac{d^{2}\phi }{dz^{2}}\right] \end{array}$$

irrespective of the signs appearing in Equation (19).

Substituting (23) and ${\left(z^{\prime }\left(x\right)\right)}^{2}$ from (20) into Equation (17) and comparing the corresponding terms one finds that the following five equations have to be satisfied simultaneously:

(25) $$\begin{array}{ccc} & & \lambda +s_{I}-p_{I}\frac{E}{C}=0\;, \end{array}$$

(26) $$\begin{array}{ccc} & & 1-m^{2}-s^{2}+s_{\mathrm{II}}-p_{\mathrm{II}}\frac{E}{C}=0\;, \end{array}$$

(27) $$\begin{array}{ccc} & & -2ms+s_{\mathrm{III}}-p_{\mathrm{III}}\frac{E}{C}=0\;, \end{array}$$

(28) $$\begin{array}{ccc} & & -2pB+s_{\mathrm{IV}}-p_{\mathrm{IV}}\frac{E}{C}=0\;, \end{array}$$

(29) $$\begin{array}{ccc} & & -p^{2}+s_{V}-p_{V}\frac{E}{C}=0\;. \end{array}$$

This set of equations point out the intimate and subtle relation that connects the parameters $p_{i}$ appearing in the z(x) function, the coupling coefficients ($s_{i}$) of the potential (23), the energy eigenvalue *E* and the parameters of the symmetrical canonical form of the confluent Heun equation (see Equation (1) with Equations (14) and (15).) Note that the first three equations above are the same as those obtained in reference [35] from the analogous treatment of the Jacobi polynomials, provided that the parameters are chosen as in Equation (16). This also means that the z(x) functions applied there can be used here too, and this will lead to a generalization of the Natanzon-class potentials obtained previously.

The simplest choice is letting only one of the $p_{i}$ parameters, say $p_{K}$ to non-zero value: in that case *E* follows directly from the corresponding equation. At the same time the term with $s_{K}$ in the potential (23) will turn into the constant $Cs_{K}$. We can see that this constant will also appear in the expression for *E*, so it represents a simple shift of the energy scale. The remaining four equations with $s_{i}$, i≠K relate the remaining four $s_{i}$ coupling coefficients to the parameters appearing in the Heun equation. Selecting $p_{K}$ also means selecting ϕ(z) and Φ(z) in Equations (18) and (20), and eventually, defining the x(z) function via Equation (7). Actually, the choices K=I and II recover the z(x) functions applied in the PI and PII class shape-invariant potentials [52], while the K=III choice leads to the uninvertible inverse x(z) function found for the implicit type PIII potential [53]. Obviously, applying these z(x) functions in the present procedure will result in potentials that are the extensions of the named Natanzon-class potentials in the sense that they contain two more potential terms (i.e., those with $s_{\mathrm{IV}}$ and $s_{V}$). Selecting $p_{\mathrm{IV}}$ or $p_{V}$ to be the only non-zero parameter leads to new z(x) functions and potentials. It is notable that while the PV case in Equation (19) leads to an explicit (and rather simple) z(x) function, in the PIV case it results in a complicated implicit x(z) function that can be expressed in terms of elliptic integrals. Table 1 and Table 2 list the potentials and the corresponding expressions for *E* in the cases discussed here.

It has to be added that in order to obtain proper state-independent quantum mechanical potentials, the reparametrization of the coupling coefficients is necessary such that they remain independent of the *n* quantum number. This *n* arises naturally in the case of Natanzon-class potentials as the degree of the Jacobi polynomial contained in the bound-state wavefunctions. In this case *n* appears in the R(z) function in Equation (1) in an expression that eventually contributes to the term proportional to $s_{I}$. In the PI case this term plays the role of *E*, so all the remaining coordinate-dependent (potential) terms will be free from *n*. In the present context this means that the *n*-dependence appears through the λ parameter.

In the PII shape-invariant class *E* arises from terms with $m^{2}$ and $s^{2}$. Furthermore, there is a potential term with coupling coefficient ms, so it is natural to reparametrize the problem such that ms=const. It is found that in this case λ can be made *n*-independent if *m* depends on *n* in a linear form, so *E* will have terms which have quadratic and inverse quadratic dependence on *n*.

In the case of the PIII (non-shape-invariant) Natanzon-class potential *E* depends on ms. Here the reparametrization was carried out by prescribing the same linear dependence of *m* on *n*, and in addition eliminating the *n*-dependence from $m^{2}+s^{2}$, which led to an *s* that is the root of a second-order algebraic equation in *n* [53]. This scenario can work in the case of the PIII type potential derived from the confluent Heun equation too.

Similar reparametrizations may be necessary in the present context if *n* appears through *p* or *B*. In the PV case *E* depends on $p^{2}$, while a potential term has pB in its coupling coefficient. It is reasonable to cancel the *p*-dependence of this term by prescribing that B∼1/p. In the PIV case if *n* appears through *B*, then all the potential terms can remain independent of *n*.

As a more general approach, further combinations of the $p_{i}$ parameters can also be made: in that case the ϕ(z) function will also be more general. There are several Natanzon-class potentials obtained in this way [32,34,54,55,56,57,58]. Most of them are of the implicit type, i.e., there is closed expression only for the inverse x(z) function. The generalizations of these Natanzon-class potentials (i.e., their extension with two extra terms) is straightforward.

It is seen that altogether there are twelve parameters: $p_{i}$, $s_{i}$, *C* and $x_{0}$, but some of these are not independent and some represent a shift of the coordinate or the energy. The $p_{i}$ parameters appear in the z(x) (or (x(z)) function, and as such they influence all the potential terms, including those arising from the Schwartzian derivative term. From (20) it is seen that only the relative magnitude of the $p_{i}$ parameters is important: their absolute magnitude is set by *C*, which also appears in the scaling of *x* (as in Equation (7)) or the energy (as in (23) and (25) to (29)). In addition, $x_{0}$ also appears through z(x) only. The $s_{i}$ parameters play the role of coupling coefficients (as in (23)) and they are related to the five parameters of the symmetric form of the Heun equation. However, they represent only four independent potential terms, as one of the potential terms reduces to a constant (as seen above), and plays the role of an energy shift.

It is worthwhile to compare the potentials to those obtained in reference [47] from the non-symmetrical canonical form of the confluent Heun equation. The link between the two approaches can be established by comparing the differential equations defining the z(x) function, i.e., (20) and (13). It also has to be noted that *z*, as the argument of the solutions of the respective equations denotes different functions in the two cases, as discussed in relation with Equation (22). Taking all these into account, (13) can be rewritten in the form of (20). One finds that the *C* constant and the ϕ(z) function of the present treatment are related to the notation of reference [47] as

(30) $$C={\left(-1\right)}^{2m_{2}}2^{2-2m_{1}-2m_{2}}\sigma^{-2}$$

and

(31) $$\phi \left(z\right)={\left(1-z\right)}^{2-2m_{1}}{\left(1+z\right)}^{2-2m_{2}}\;.$$

The $p_{i}$ parameter settings corresponding to the potentials described in reference [47] are listed in Table 3. It is seen that direct correspondence between the potentials with the simplest choice of the parameters exists for the situations in which ϕ(z) is expressed as the power of $\left(1-z^{2}\right)$, i.e., when $m_{1}=m_{2}=0$, 1/2 and 1. In the present treatment these cases correspond to the z(x) functions of the PI and PII class shape-invatiant potentials [52] and to the new PV case with only $p_{V}$ chosen non-zero.

To conclude this Section, we can establish that the potentials generated from the symmetrical canonical form of the Heun equation represent an extension of the Natanzon-class potentials in two respects. First, there are two more parameters ($p_{\mathrm{IV}}$ and $p_{V}$) that appear in the z(x) function, so there are more choices for generating appropriate coordinate transformations. Second, there are also two more parameters ($s_{\mathrm{IV}}$ and $s_{V}$) playing the role of coupling coefficients, so the potentials can be extended with two more independent terms. One also has to add that the present scheme is not complete without the explicit construction of the solutions in terms of the Hc(p,α,γ,δ,σ;z) fuctions. Normally, the bound-state solutions are obtained by prescribing physical boundary conditions on the mathematical solutions of the Schrödinger equation. In the case of Natanzon-class potentials this meant that the hypergeometric function was reduced to a Jacobi polynomial as the consequence of the a=−n or b=−n choice, introducing the *n* quantum number. This quantum number is expected to appear only in the $E=E_{n}$ energy eigenvalues, which usually requires the reparametrization of the potential. The structure of the solutions of the Heun equation is much less known, so at the moment the physical solutions cannot be expressed explicitly. Neverthleless, the construction presented here simplifies finding bound-state solutions once the explicit form of the Hc(z) functions is available. The present results also serve as a firm platform for the discussion of the PT-symmetric version of these potentials in their abstract form. This is the subject of the ensuing Section.

## 4. Implementing PT Symmetry to the Potentials

The PT symmetry requirement $V^{*}\left(-x\right)=V\left(x\right)$ introduces strong constraints on the potential and the parameters appearing in them. In practice, this requirement implies that the even and odd potential components have to be real and imaginary, respectively. In order to construct a PT-symmertic potential, it can be useful to have definite PT-symmetry property of its ingredients. If z(x) and ϕ(z(x)) are of this type, i.e., if they are PT-even or PT-odd, then the construction procedure is simplified significantly. This observation was the motivation behind considering the symmetrical canonical form of the confluent Heun equation instead of the non-symmetrical form. In this Section we investigate the transformation properties of the potentials generated from Section 3 with respect to the parity (P) and PT operations. The formulas derived there also imply strong correlations between the PT property of z(x), ϕ(z(x)) and V(x). In the discussion we make use of some of the results obtained in reference [35] for the similar treatment of Natanzon-class potentials.

In order to implement PT symmetry to a potential, first it is necessary to check whether its domain of definition is suitable for the application of the P operation. This operator interchanges $X_{0}+x$ with $X_{0}-x$, where $X_{0}$ is the center of reflection. Since a coordinate shift has no influence on the physical properties of a system, $X_{0}$ can be chosen to be $X_{0}=0$ in general. If *x* is defined on the whole real axis (as it is the case for the potentials PI(z(x)=isinh(ax)), PII(z(x)=tanh(ax)) or PV(z(x)=iax) in Table 2), then no further change is necessary for implementing PT symmetry. The situaton is similar if V(x) is defined on a finite domain (as is the case for PI(z(x)=cos(ax)), PII(z(x)=itan(ax)) or PV(z(x)=ax) in Table 2). In this case perhaps $X_{0}\neq 0$ has to be chosen in order to position V(x) properly. (z(x)=sin(ax) would correspond to such a situation with $X_{0}=\pi /2$ in the PI case, but it would not lead to an essentially new potential.) The situation is rather different for potentials defined on x∈[0,∞) (e.g., PI(z(x)=cosh(ax), PII(z(x)=coth(ax) or PIII in Table 2). These potentials typically have a singularity at x=0 which prevents their extension to x<0. However, in the case of PT symmetry, the singularity can be circumvented by applying an imaginary coordinate shift $x_{0}=i\epsilon$, which arise from Equation (7) as a constant of integration. Changing *x* to x+iϵ breaks P symmetry, but respects PT symmetry, as PT(x+iϵ)=−(x+iϵ). The z(x) functions defined this way lose (P) parity, but exhibit definite PT parity. z(x)=cosh(x+iϵ) will be PT-even, while z(x)=coth(x+iϵ) will be PT-odd. The potentials will be finite at x=0, so they can be extended to x<0. There are potentials that have to be defined on PT-symmetric trajectories in the complex *x* plane in order to generate solutions with appropriate boundary conditions, however, we are not going to discuss them here.

### 4.1. Parity Considerations

Assuming z(x) has definite parity, i.e., Pz(x)≡z(−x)=±z(x) one finds that the potential terms originating from the Schwartzian derivative will be even function of *x*. According to Equation (20) the parity of the ϕ(z(x)) function depends on the $p_{i}$ parameters and on the parity of z(x):

(32) $$\phi (z(x))\mathrm{is}\left\{\begin{array}{cc} \mathrm{even} & \mathrm{if}\;z(x)\;\mathrm{is}\;\mathrm{even}\\ \mathrm{even} & \mathrm{if}\;z\left(x\right)\mathrm{is}\;\mathrm{odd}\;\mathrm{and}\;p_{\mathrm{III}}=0\;,p_{\mathrm{IV}}=0\\ \mathrm{odd} & \mathrm{if}\;z\left(x\right)\mathrm{is}\;\mathrm{odd}\;\mathrm{and}\;p_{I}=p_{\mathrm{II}}=p_{V}=0\\ \mathrm{indefinite} & \mathrm{if}\;z(x)\mathrm{has}\;\mathrm{no}\;\mathrm{definite}\;\mathrm{parity} \end{array}\right.$$

The parities of z(x) and that of ϕ(z(x)), are also linked by the relation (19). This relation implies that $z^{\prime }\left(x\right)$ and ${\left[\phi \left(z\left(x\right)\right)\right]}^{1/2}$ have the same or opposite parity, depending on the sign in Equation (19). This means that the parity of z(x) and ${\left[\phi \left(z\left(x\right)\right)\right]}^{1/2}$ have to be the opposite or the same. When z(x) is even, then $z^{\prime }\left(x\right)$ has to be odd, i.e., it has to vanish at x=0. In this case opposite signs have to be chosen in (19) for x>0 and x<0. This also implies z(0)=1, what is confirmed by the cases listed in Table 1. If z(x) is odd, then $z^{\prime }\left(x\right)$ is even, so the same signs have to be taken for x>0 and x<0.

There are choices of ϕ(z) that lead to a contradiction when substituted into (19). As discussed in reference [35], $\phi \left(z\right)=z^{2}$ leads to a situation in which the parity of $z^{\prime }\left(x\right)$ has to be the same as the parity of ±z(x). This occurs when the only non-zero $p_{i}$ are $p_{I}=-1$ and $p_{\mathrm{II}}=1$, corresponding to the DKV (Dutt-Khare-Varshni) potential [32,56,59]. In this case the contradiction is resolved by the fact that $z\left(x\right)={\left[1+D\exp\left(-2ax\right)\right]}^{1/2}$ has indefinite parity, so the initial assumption that z(x) has definite parity is invalid.

Equation (24) also leads to a prescription concerning the parity conditions of z(x) and ϕ(z(x)). If we assume that z(x) has definite parity, then the left side of this equation has to be an even function of *x*. The parity of the right side, however, depends on that of z(x) and ϕ(z(x)). If the latter is even, then there is no contradiction. However, assuming that ϕ(z(x)) is odd, which also implies that z(x) has to be odd (see (32)), one finds that the left side of (24) is odd. This can occur when only $p_{\mathrm{III}}$ and/or $p_{\mathrm{IV}}$ are non-zero.

### 4.2. PT-Parity Considerations

As it was discussed at the introduction of this Section, the implementation of PT symmetry requires extending the domain of definition of the potentials to x<0 by appropriate real or imaginary coordinate shifts. It is also important to establish that since $C^{1/2}$ plays the role of a scaling factor of the coordinate, a general complex *C* would be incompatible with PT symmetry. The scaling factor is real or imaginary, depending on whether *C* is positive or negative.

In what follows we assume that the z(x) function has definite PT-parity $\mathrm{PT}z\left(x\right)\equiv z^{*}\left(-x\right)=\pm z\left(x\right)$. Under these conditions the PT transformation properties of ϕ(z(x)) will be determined by

(33) $$\mathrm{PT}\phi \left(z\left(x\right)\right)=p_{I}^{*}\left(1-z^{2}\left(x\right)\right)+p_{\mathrm{II}}^{*}\pm p_{\mathrm{III}}^{*}z\left(x\right)\pm p_{\mathrm{IV}}^{*}z\left(x\right)\left(1-z^{2}\left(x\right)\right)+p_{V}^{*}{\left(1-z^{2}\left(x\right)\right)}^{2}\;.$$

This leads to the following conditions:

(34) $$\phi (z(x))is\left\{\begin{array}{cc} \mathrm{PT}\text{-}\mathrm{even} & \mathrm{if}\;z\left(x\right)\;\mathrm{is}\;\mathrm{PT}\text{-}\mathrm{even},\mathrm{all}\;p_{i}\;\mathrm{real}\\ \mathrm{PT}\text{-}\mathrm{even} & \mathrm{if}\;z\left(x\right)\;\mathrm{is}\mathrm{PT}\text{-}\mathrm{odd},\;p_{I},p_{\mathrm{II}},p_{V}\;\mathrm{real},p_{\mathrm{III}},p_{\mathrm{IV}}\;\mathrm{imaginary}\\ \mathrm{PT}\text{-}\mathrm{odd} & \mathrm{if}\;z\left(x\right)\;\mathrm{is}\mathrm{PT}\text{-}\mathrm{odd},\;p_{I}=p_{\mathrm{II}}=p_{V}=0,p_{\mathrm{III}},p_{\mathrm{IV}}\;\mathrm{real}\\ \mathrm{indefinite} & \mathrm{if}\;z(x)\;\mathrm{has}\;\mathrm{no}\;\mathrm{definite}\;\mathrm{PT}\text{-}\mathrm{parity} \end{array}\right.$$

Again, Equation (24) represents a constraint on the PT parity of z(x) and ϕ(z(x)). If z(x) has definite PT parity, then the left side of this equation is PT-even, so all terms on the right side also have to have this property. One finds that this is possible only if ϕ(z(x)) is PT-even. Whenever ϕ(z(x)) is PT-odd, the z(x) function arising from the integration of (19) will have indefinite PT parity. We conclude that PT-odd z(x) functions cannot lead to PT-symmetric potentials if ϕ(z(x)) is PT-odd.

This finding simplifies identifying the conditions for the PT symmetry (PT-evenness) of V(x). These are:

(35) $$V^{*}\left(-x\right)=V\left(x\right)\mathrm{if}\left\{\begin{array}{c} z\left(x\right)\mathrm{is}\;\mathrm{PT}\text{-}\mathrm{even},\;\mathrm{all}\;s_{i}\mathrm{real}\\ z\left(x\right)\mathrm{is}\;\mathrm{PT}\text{-}\mathrm{odd},\;s_{I},s_{\mathrm{II}},s_{V}\;\mathrm{real},\;s_{\mathrm{III}},s_{\mathrm{IV}}\mathrm{imaginary} \end{array}\right.$$

These conditions have to be combined with those for the $p_{i}$ parameters guaranteeing the PT-evenness of ϕ(z(x)), i.e., the first two lines of Equation (34).

The considerations presented are illustrated by the examples listed in Table 1 and Table 2. It is also worthwhile to inspect Equations (25) to (29) in light of the restrictions imposed on the $p_{i}$ and $s_{i}$ parameters by PT symmetry, because they are indicative with respect to the question whether *E* is real or complex, i.e., whether PT symmetry can be broken or not. In what follows we consider those $p_{i}$ combinations, which led to exactly solvable PT-symmetric potentials.

If z(x) is PT-even, then, according to Equations (33) and (35) all the $p_{i}$ and $s_{i}$ parameters have to be real. This means that *E* can be complex only in case one or more of the parameters λ, *m*, *s*, *B* and *p* are complex.

All the PI-type potentials appearing in Table 1 and Table 2 are derived from PT-even z(x) functions: isinh(ax), cosh(ax+iϵ), cos(ax). (In the second case the imaginary coordinate shift is needed to avoid the singularity of V(x) at x=0.) Furthermore, in these cases all the $p_{i}$ parameters besides $p_{I}$ are zero, which reduce further the possible parameter settings. Condition (29), for example, prescribes that *p* is either real or imaginary, depending on the sign of $s_{V}$. Condition (28) forces *B* also to real or imaginary valuse, depending on whether *p* is real or imaginary. Conditions (26) and (27) imply that both ${\left(m+s\right)}^{2}=s_{II}+s_{III}+1$ and ${\left(m-s\right)}^{2}=s_{II}-s_{III}+1$ have to be real, i.e., m+s and m−s have to be either real or imaginary, depending on the relative magnitude of $s_{II}$ and $s_{III}$. From condition (25) it is seen that *E* can be complex only when λ is complex. In the case of the shape-invariant Natanzon-class Scarf II, Scarf I and generalized Pöschl–Teller potentials λ depends on *m*, as seen from (16), so *E* is complex here if *m* is complex. Such a relation between λ and the other parameters is not known for the general potentials discussed here. It is expected that further analysis of normalizability or the explicit form of the solutions may introduce such a relation.

The PII-type potentials are examples for both PT-even (z(x)=itan(ax)) and PT-odd z(x) (z(x)=tanh(ax), z(x)=coth(ax+iϵ)) functions. In both cases condition (25) precribes that λ is real. In the case of the shape-invariant Natanzon-class PII-type potentials (Rosen–Morse II, II, Eckart) this also meant that *m* is real, so $m^{2}$ is also real, as is $s^{2}$, according to condition (27). This simple analysis proved [35] that according to condition (26) these potentials can have only pure real energy spectrum, so the breaking of PT symmetry cannot occur for them. In the present situation the relation of λ and *m* is not known, so one cannot reach a similar conclusion concerning the energy spectrum of PII-type potentials. However, one can establish that for z(x)=tanh(ax) and coth(ax+iϵ)*p* and *B* are simultanously real or imaginary, depending on the sign of $s_{V}$, while for z(x)=itan(ax) they have opposite character. In the latter case ms is also imaginary.

The PT-symmetric version of the PIII potential [53] has not been discussed yet. It is an implicit potential constructed from an implicit x(z) function defined for x≤0 and z≤0 as

(36) $$\begin{array}{ccc} x+i\epsilon & = & C^{-1/2}\left[\tanh^{-1}\left(z^{1/2}\right)-\tan^{-1}\left(z^{1/2}\right)\right] \end{array}$$

(37) $$\begin{array}{ccc} & = & C^{-1/2}\sum_{k=0}^{\infty }\frac{z^{2k+3/2}}{2k+3/2}\;, \end{array}$$

where (37) follows from the series expansion of (36). For a PT-symmetric extension to x<0 and for avoiding the singularity at x=0, the introduction of an imaginary coordinate shift x→x+iϵ could be used. However, the particular form of x(z), and especially (37) does not seem suitable to define a z(x) function with definite PT-symmetric transformation property that would be imaginary (iϵ) at x=0. So we conclude that the PT-symmetrization of the PIII potential, and thus that of its generalisation in the present scheme, may not be possible.

Due to the non-trivial implicit form of the x(z) function, we do not discuss here the PT symmetry of the PIV class potential either. The PV class is, however, rather straightforward: it leads to a PT-even (iax) and a PT-odd (ax) z(x) function (see Table 1 and Table 2). In the former case λ, $m^{2}+s^{2}$, ms and pB have to be real, while in the latter case ms and pB have to be imaginary.

There are several PT-symmetric potentials derived from z(x) functions with more than one non-zero $p_{i}$ parameter. In some cases the $p_{i}$ parameters are fixed, while in some others at least one of them is tunable, introducing one more free model parameter.

The Dutt-Khare-Varshni (DKV) potential was introduced [32] as a conditionally solvable potential meaning that it contained potential terms that had fixed coupling coefficient. This potential was later recognised [56] as a Natanzon-class potential, and the potential terms with fixed coupling coefficient were identified as those originating from the Schwartzian derivative. It can be recovered from the the present approach by making the choice $p_{I}=-1$, $p_{II}=1$, which leads to $\phi \left(z\right)=z^{2}$. Substituting this into Equation (19) and integrating it one obtains $z\left(x\right)={\left[1+D\exp\left(-2ax\right)\right]}^{1/2}$ for $C=a^{2}$ and with a particular choice of the signs and the integration constant. This z(x) function has no definite parity, as has been discussed previously. The PT-symmetric version of the DKV potential was introduced in reference [59]. There the formalism was changed by selecting $C=-a^{2}$ instead of $C=a^{2}$, and this defined a z(x) function with definite, even PT-parity.

The same z(x) function can also be applied to construct the generalization of the PT-symmetric DKV potential to the present scheme. With the choice $p_{I}=-1$, $p_{II}=1$ the conditions (25) to (29) lead to the following prescriptions for the model parameters. Since all the $s_{i}$ are real, one finds that $\lambda -m^{2}-s^{2}$ and ms are real, so $\lambda -{\left(m\pm s\right)}^{2}$ is also real, furthermore, *p* and *B* are simultaneously real or imaginary, depending on the sign of $s_{V}$. In the Natanzon limit (p=0) λ is real and is related to *m* by (16), so the PT-symmetric DKV potential can have only real energy eigenvalues.

Another non-trivial PT-symmetric Natanzon-class potentials has been discussed in reference [34]. The extension of this potential is also possible by introducing two new terms, i.e., those with the parameters $s_{IV}$ and $s_{V}$ in Equation (23). For this the same z(x) (rather, x(z)) function has to be used as in reference [34]. This is obtained by the choice $p_{I}=1$ and $p_{II}=\delta$, so the relevant ϕ(z) function will be $\phi \left(z\right)=\delta +1-z^{2}$. Obviously, δ=0 will recover the PI-type potentials (depending on whether $C=a^{2}$ or $C=-a^{2}$), while the PII type potentials are obtained by taking the limit δ→∞ while keeping C/δ constant. Furthermore, for δ=−1 the DKV potential is obtained as a special limit. In reference [34] the case $C=-a^{2}$, δ≥0 was considered in detail, which contained the Scarf II and the Rosen–Morse I potentials as special PI-type and PII-type limits. It was found that the implicit z(x) function was expressed in terms of the inverse tan and tanh functions, and that z(x) is PT-even. This implies that all the $s_{i}$ parameters have to be real. Substituting these, $p_{I}$, $p_{II}$ and *C* in Equations (25) to (29) one finds that *p* and *B* are simultaneously either real or imaginary, ms is real, while *E* and ${\left(m\pm s\right)}^{2}$ real or complex depending on whether λ is real or complex.

In reference [34] it was found that combining Equations (25) to (27) a quartic algebraic equation is obtained for *m*, and solving it results in the $E_{n}$ energy eigenvalues. It was also found that by tuning a parameter corresponding to $s_{III}$ the pairs of real energy eigenvalues turn into complex conjugate pairs such that the complexification starts from the ground state as $s_{III}^{2}$ is increased from zero. This process was identified as the “gradual” breaking of PT symmetry, which was distinct from the "sudden" mechanism characterising most known exactly solvable Natanzon-class potentials, where the complexification of the energy eigenvalues occurred at the same parameter value for each level. The $s_{III}=0$ case was also identified as the Ginocchio potential [54].

In the present scheme the relation of λ to the other model parameters is not known, so it is not possible to find out when *E* is real or complex. For this further conditions have to be established by the inspection of the general solutions from the point of view of boundary conditions.

The generalized Ginocchio potential [55] was also considered in the PT-symmetric setting [33]. This potential was originally defined on the positive real *x* axis, as it exhibits a singularity at x=0, so in order to extend it to x<0 an imaginary coordinate shift had to be applied. It was found that the breakdown of PT symmetry occurs via the “sudden” mechanism, i.e., the complexification of the energy eigevalues occurs at the same parameter value for all the levels. In the present scheme this potential is obtained from the parametrization $p_{I}=\gamma^{2}-1$, $p_{II}=p_{III}=2$ and $C=4\gamma^{2}$. The corresponding z(x) function is obtained implicitly in terms of inverse tan and tanh functions and it is PT-even. The energy eigenvalues of the generalized Ginocchio potential are obtained from Equations (25) to (27). The energy eigenvalues are obtained by solving a quadratic algebraic equation for m+s. The complexification of the energy eigenvalues is obtained from shifting m−s form real to imaginary values.

The extension of this potential to the case obtained form the confluent Heun equation means using the same z(x) function and adding two terms (those with $s_{IV}$ and $s_{V}$ in (23)). In this case *p* and *B* are simultaneously real or imaginary, according to Equations (28) and (29).

One can inspect the potentials derived from the non-symmetrical canonical form of the confluent Heun equation in reference [47]. For this the $p_{i}$ parameters listed in Table 3 have to be substituted into Equations (25) to (29), and the relevant z(x) (or x(z)) functions have to be determined from Equation (19). It can be then determined whether the resulting z(x), ϕ(z(x)) and V(x) functions are compatible with the requirements of PT symmetry. This has to be established in each case separately.

In addition to the PT transformation properties of the potential, it is also instructive to inspect those of the solutions (22). Before specifying them to the PT-symmetric setting, some general remarks are in order. The singular points of the confluent Heun equation deserve special attention. In (22) they occur at z=±1 and z→±∞. In the latter case the exponential factor exp(−pz(x)) can lead to infinite expressions. In order to avoid these the condition limx→±∞Re(pz(x))>0 has to be satisfied whenever the z(x) function tends to infinity asymptotically. This can exclude odd z(x) functions in certain situations.

Solutions that are eigenfunctions of the PT transformation belong to real energy eigenvalues and unbroken PT symmetry, while the solutions belonging to complex conjugate energy eigenvalues and broken PT symmetry are connected by the PT operator. The transformation property of the pre-factor of the solution can be established in a simple way if that of the z(x) function is known. Remember also that the ϕ(z(x)) function (20) appearing in this pre-factor has to be PT-even in order to allow for the construction of PT-symmetric potentials. The structure of the confluent Heun function Hc is not known a priori, however, assuming that it can be expressed in power series form or as the linear combination of (confluent) hypergeometric functions, its behaviour under complex conjugation (the T operation) can also be established.

Considering all these circumstances, the PT transform of the unnormalized (22) solution has the form

(38) $$\begin{array}{ccc} \mathrm{PT}\psi (x) & ≃ & \phi^{\frac{1}{4}}\left(z\left(x\right)\right)\exp\left(-p^{*}z\left(x\right)\right){\left(1+z\left(x\right)\right)}^{\frac{1}{2}\left(m^{*}-s^{*}\right)}{\left(1-z\left(x\right)\right)}^{\frac{1}{2}\left(m^{*}+s^{*}\right)}\\ & & \times Hc(p^{*},-B^{*}+m^{*}+1,m^{*}+s^{*}+1,m^{*}-s^{*}+1,\\ & & \;\;\;\;\;\lambda^{*}-m^{*}\left(m^{*}+1\right)-2p^{*}\left(B^{*}-m^{*}-s^{*}-1\right);\frac{1}{2}\left(1-z\left(x\right)\right)\; \end{array}$$

for PT-even z(x) functions, while

(39) $$\begin{array}{ccc} \mathrm{PT}\psi (x) & ≃ & \phi^{\frac{1}{4}}\left(z\left(x\right)\right)\exp\left(p^{*}z\left(x\right)\right){\left(1-z\left(x\right)\right)}^{\frac{1}{2}\left(m^{*}-s^{*}\right)}{\left(1+z\left(x\right)\right)}^{\frac{1}{2}\left(m^{*}+s^{*}\right)}\\ & & \times Hc(p^{*},-B^{*}+m^{*}+1,m^{*}+s^{*}+1,m^{*}-s^{*}+1,\\ & & \;\;\;\;\;\lambda^{*}-m^{*}\left(m^{*}+1\right)-2p^{*}\left(B^{*}-m^{*}-s^{*}-1\right);\frac{1}{2}\left(1+z\left(x\right)\right)\; \end{array}$$

if z(x) is PT-odd. The next question is whether these solutions are identical (up to a numerical factor) with (22) or not. Since the argument of the confluent Heun function changed in (39), direct comparison is possible only for (38). It is straightforward to show that (22) and (38) match if all the parameters *p*, *m*, *s*, *B* and λ are real. Remembering also that for PT-even z(x) functions the PT symmetry of V(x) implies that all the $p_{i}$ parameters in ϕ(z(x)) in (20) and all the $s_{i}$ parameters in (23) have to be real (see Equations (34) and (35)). Combining all this with the conditions (25) to (29) one can easily notice that in this case the energy eigenvalues can take on only real value. This situation corresponds to unbroken PT symmetry.

There are also further solutions of the confluent Heun equation [36] that can be compared with the solutions (38). It is known that if Hc(p,α,γ,δ,σ;y) is the solution of this differential equation, then so are the following functions:

(40) $$\begin{array}{ccc} & & y^{1-\gamma }Hc\left(p,-\alpha +\gamma -1,2-\gamma ,\delta ,\sigma +\left(-4p+\delta \right)\left(\gamma -1\right);y\right)\;, \end{array}$$

(41) $$\begin{array}{ccc} & & {\left(y-1\right)}^{1-\delta }Hc\left(p,-\alpha +\delta -1,\gamma ,2-\delta ,\sigma -\gamma \left(1-\delta \right);y\right)\;, \end{array}$$

(42) $$\begin{array}{ccc} & & \exp(-4py)Hc(-p,\alpha -\gamma -\delta ,\gamma ,\delta ,\sigma -4p\gamma ;y)\;. \end{array}$$

The combination of these transformations can lead to further solutions of the confluent Heun equation. The question whether they are linearly independent from each other and Hc(p,α,γ,δ,σ;y) is non-trivial, and is the subject of investigations [36].

Substituting these functions into the general solution (22) of the Schrödinger equation with the parameters (21) and y=(1−z(x))/2 one can ask whether the resulting solutions can take the form of (38). If any of the functions (40) to (42) are identical with the original Hc(p,α,γ,δ,σ;y) function, then we again obtain the situation with unbroken PT symmetry and real energy eigenvalues *E*. On the contrary, if they are linearly independent, then the two solutions will belong to two complex conjugate energy eigenvalues corresponding to the breakdown of PT symmetry.

The comparison of (38) and the solution with (40) leads to the following reults: *p* and m=−s are real, while *B* and λ can be complex with correlated imaginary components $\lambda_{I}=2pB_{I}$ and $B_{R}=m+1$. Combining these with the requirement that all the $p_{i}$ and $s_{i}$ parameters are real one finds that complex energy eigenvalues can be obtained only when $p_{I}$ and $p_{IV}$ are non-zero, while all the other $p_{i}$ are zero. In this case λ and *B* are complex. In all the other cases *E* is real, and so are λ and *B*. Essentially the same results are found taking the solution with (41) with the difference that now m=s is valid. For the solutions with (42) one obtains that *p* is imaginary, *B*, *s* and m=−1 are real, while λ can be complex with $\lambda_{I}=-2iB_{I}p$. Complex *E* can occur only when only $p_{I}$ is non-zero, and all the other $p_{i}$ are zero, i.e., in the PI case indicated in Table 1 and Table 2. In any other case *E* is real, and *B* has to be zero.

There is also another solution of the confluent Heun equation the argument of which is 1−y rather than *y*:

(43) Hc(−p,α,δ,γ,σ+4pα;1−y).

Note the reverse order of γ and δ. Substituting this function into (22) with the parameters (21) and y=(1−z(x))/2 the comparison with (39) becomes possible. It turns out in this case that *p* and *s* are imaginary, *m* and *B* are real, while λ can be complex with $\lambda_{I}=-4ip\left(B-m-1\right)$. Combining these with the requirement for the $p_{i}$ and $s_{i}$ parameters for PT-odd z(x) functions (see Equations (34) and (35)) and analysing conditions (25) to (29) one finds that the energy eigenvalue *E* is real, except when only $p_{I}$ is non-zero, i.e., in the PI case. In the former case λ is also real, and B=m+1 has to hold.

## 5. Summary

In the present study we discussed quantum mechanical potentials originating from the confluent Heun equation and discussed conditions under which they can satisfy the PT invariance requirement. This work was motivated by our earlier systematic study in which we discussed the construction of the PT-symmetric version of general Natanzon-class potentials. The solutions of these potentials are written in terms of the hypergeometric function, which reduce to Jacobi polynomials for bound-state solutions. A main conclusion of that study was that the formalism based on the differential equation of the Jacobi polynomials is more suitable for implementing PT symmetry to the potentials. This is because the z(x) function that controls the variable transformation and plays a central role in deriving solvable potentials and their solutions can be cast in a PT-symmetric form from the beginning, and this facilitates the implementation of the PT invariance requirement. A similar conclusion was reached in the present study too, as it turned out that setting out from the symmetrical canonical form of the confluent Heun differential equation is more suitable for the reasons mentioned before.

The confluent Heun equation was a natural choice for generalizing the formalism beyond the Natanzon potential class, because it can be reduced to the hypergeometric equation in a natural way. A major difference with respect to the case of Natanzon-class potentials and their solutions in terms of hypergeometric functions (Jacobi polynomials) is that the explicit form of the solutions of the Heun differential equation and its confluent variants is not known in general. For this reason the construction of the bound-state solutions with proper boundary conditions and energy eigenvalues is not possible. Nevertheless, the general properties of potentials derived from these equations can be established. A study of this kind was already performed for the confluent Heun equation in reference [47]. There the non-symmetrical canonical form of this differential equation was considered. (It is notable that a less detailed analysis of this question was already presented fifty years ago, together with potentials related to other types of the Heun equation too; see reference [48].) Since the symmetrical and non-symmetrical canonical form of the confluent Heun equation are connected by a variable transformation, different z(x) functions are used to derive solvable potentials from them. We also discussed how the potentials derived in reference [47] can be related to those studied here.

We identified the general form of potentials derived from the symmetrical canonical form of the confluent Heun equation, and found that they depend on twelwe parameters. Five of these ($p_{i}$) control the z(x) variable transformation, four ($s_{i}$) play the role of individually tunable coupling coefficients of potential terms, while three (*C*, $x_{0}$ and one of the $s_{i}$) contribute to scaling and shifting the coordinate and the energy. Further potential terms with fixed coupling coefficients can also originate from the Schwartzian derivative that depends on the z(x) variable transformation function and the parameters appearing in it.

A major result of this analysis is the set of Equations (25) to (29), which establish a link between the parameters of the model with the energy eigenvalue *E* and the parameters appearing in the confluent Heun equation. The analysis of these equations allows a deep insight into the possible structure of the potential, its solutions and its allowed energy eigenvalues. This applies also to the prescriptions under which the potential can be cast in PT-symmetric form from, furthermore, also to the conditions under which the complexification of the energy eigenvalues, i.e., the breakdown of PT symmetry can occur. We applied these considerations also to the the solutions of the Schrödinger equation and inspected whether they are the eigenfunctions of the PT operator. We extended this analysis also to the transformed forms of the confluent Heun functions. All these analyses were possible without the explicit knowledge of the confluent Heun functions.

These results may inspire further study of the solutions of the Heun type differential equations. Any new finding, i.e., a closed solution for the confluent Heun function can directly be combined with the results of the present study. These efforts can ultimately lead to the explicit knowledge of new solvable potential families.

## Figures and Tables

**Table 1:** Ingredients of potentals generated by chosing a single $p_{i}$ parameter as non-zero, $p_{K}=1$.

	${\left(z^{\prime }\left(x\right)\right)}^{2}$	*C*	ϕ(z)	z(x)	x∈	*E*
PI	$C(1-z^{2})$		$\left(1-z^{2}\right)$	
		$-a^{2}$	$\cosh^{2}\left(ax\right)$	isinh(ax)	(−∞,∞)	$-a^{2}\left(\lambda +s_{I}\right)$
		$-a^{2}$	$-\sinh^{2}\left(ax\right)$	cosh(ax)	[0,∞)	$-a^{2}\left(\lambda +s_{I}\right)$
		$a^{2}$	$\sin^{2}\left(ax\right)$	cos(ax)	$\left[-\frac{\pi }{2a},\frac{\pi }{2a}\right]$	$a^{2}\left(\lambda +s_{I}\right)$
PII	$C{\left(1-z^{2}\right)}^{2}$		1	
		$a^{2}$	1	tanh(ax)	(−∞,∞)	$a^{2}\left(1-m^{2}-s^{2}+s_{\mathrm{II}}\right)$
		$a^{2}$	1	coth(ax)	[0,∞)	$a^{2}\left(1-m^{2}-s^{2}+s_{\mathrm{II}}\right)$
		$-a^{2}$	1	itan(ax)	$\left[-\frac{\pi }{2a},\frac{\pi }{2a}\right]$	$-a^{2}\left(1-m^{2}-s^{2}+s_{\mathrm{II}}\right)$
PIII	$C\frac{{\left(1-z^{2}\right)}^{2}}{z}$
		*C*	*z*	impl. [53]	[0,∞)	$-2msC+Cs_{\mathrm{III}}$
PIV	$C\frac{\left(1-z^{2}\right)}{z}$
		*C*	$z(1-z^{2})$	impl.		$-2pBC+Cs_{\mathrm{IV}}$
PV	*C*		${\left(1-z^{2}\right)}^{2}$	
		$a^{2}$	$1-a^{2}x^{2}$	ax	$\left[-\frac{1}{a},\frac{1}{a}\right]$	$-a^{2}\left(p^{2}-s_{V}\right)$
		$-a^{2}$	$1+a^{2}x^{2}$	iax	(−∞,∞)	$a^{2}\left(p^{2}-s_{V}\right)$

**Table 2:** Potentals corresponding to the choices made in Table 1. The potential terms are indicated individually, including those (in square brackets) obtained from the Schwartzian derivative.

	$\left[-\frac{z^{\prime \prime \prime }}{2z^{\prime }}+\frac{3}{4}{\left(\frac{z^{\prime \prime }}{z^{\prime }}\right)}^{2}\right]+\frac{Cs_{I}\left(1-z^{2}\right)}{\phi (z)}+\frac{Cs_{\mathrm{II}}}{\phi (z)}+\frac{Cs_{\mathrm{III}}z}{\phi (z)}+\frac{Cs_{\mathrm{IV}}z\left(1-z^{2}\right)}{\phi (z)}+\frac{Cs_{V}{\left(1-z^{2}\right)}^{2}}{\phi (z)}$
PI
	$\left[\frac{a^{2}}{4}-\frac{3a^{2}}{4\cosh^{2}\left(ax\right)}\right]-s_{I}a^{2}-a^{2}\frac{m^{2}+s^{2}-1}{\cosh^{2}\left(ax\right)}-2msa^{2}i\frac{\sinh(ax)}{\cosh^{2}\left(ax\right)}-2pBa^{2}i\sinh\left(ax\right)-p^{2}a^{2}\cosh^{2}\left(ax\right)$
	$\left[\frac{a^{2}}{4}+\frac{3a^{2}}{4\sinh^{2}\left(ax\right)}\right]-s_{I}a^{2}+a^{2}\frac{m^{2}+s^{2}-1}{\sinh^{2}\left(ax\right)}+2msa^{2}\frac{\cosh(ax)}{\sinh^{2}\left(ax\right)}-2pBa^{2}\cosh\left(ax\right)+p^{2}a^{2}\sinh^{2}\left(ax\right)$
	$\left[-\frac{a^{2}}{4}+\frac{3a^{2}}{4\sin^{2}\left(ax\right)}\right]+s_{I}a^{2}+a^{2}\frac{m^{2}+s^{2}-1}{\sin^{2}\left(ax\right)}+2msa^{2}\frac{\cos(ax)}{\sin^{2}\left(ax\right)}+2pBa^{2}\cos\left(ax\right)+p^{2}a^{2}\sin^{2}a\left(x\right)$
PII
	$\left[a^{2}\right]-\frac{\lambda a^{2}}{\cosh^{2}\left(ax\right)}+s_{\mathrm{II}}a^{2}+2msa^{2}\tanh\left(ax\right)+2pBa^{2}\frac{\sinh(ax)}{\cosh^{3}\left(ax\right)}+\frac{p^{2}a^{2}}{\cosh^{4}\left(ax\right)}$
	$\left[a^{2}\right]+\frac{\lambda a^{2}}{\sinh^{2}\left(ax\right)}+s_{\mathrm{II}}a^{2}+2msa^{2}\coth\left(ax\right)-2pBa^{2}\frac{\cosh(ax)}{\sinh^{3}\left(ax\right)}+\frac{p^{2}a^{2}}{\sinh^{4}\left(ax\right)}$
	$\left[-a^{2}\right]+\frac{\lambda a^{2}}{\cos^{2}\left(ax\right)}-s_{\mathrm{II}}a^{2}-2msa^{2}i\tan\left(ax\right)-2pBa^{2}i\frac{\sin(ax)}{\cos^{3}\left(ax\right)}-\frac{p^{2}a^{2}}{\cos^{4}\left(ax\right)}$
PIII
	$\left[\frac{3Cz}{16}+\frac{9C}{8z}-\frac{5C}{16z^{3}}\right]-\frac{C}{z}\lambda \left(1-z^{2}\right)+C\frac{\left(m^{2}+s^{2}-1\right)}{z}+Cs_{\mathrm{III}}-2pBC\left(1-z^{2}\right)+C\frac{p^{2}{\left(1-z^{2}\right)}^{2}}{z}$
PIV
	$\left[-\frac{3C}{16z}+\frac{3C}{4z(1-z^{2})}-\frac{5C}{z^{3}\left(1-z^{2}\right)}\right]-\frac{C\lambda }{z}+C\frac{\left(m^{2}+s^{2}-1\right)}{z(1-z^{2})}-\frac{2msC}{1-z^{2}}+Cs_{\mathrm{IV}}+C\frac{p^{2}\left(1-z^{2}\right)}{z}$
PV
	$\left[0\right]-\frac{\lambda a2}{1-a^{2}x^{2}}+a^{2}\frac{\left(m^{2}+s^{2}-1\right)}{{\left(1-a^{2}x^{2}\right)}^{2}}+2msa^{3}\frac{x}{{\left(1-a^{2}x^{2}\right)}^{2}}+2pBa^{3}\frac{x}{1-a^{2}x^{2}}+s_{V}a^{2}$
	$\left[0\right]+\frac{\lambda a^{2}}{1+a^{2}x^{2}}-a^{2}\frac{\left(m^{2}+s^{2}-1\right)}{{\left(1+a^{2}x^{2}\right)}^{2}}-2msa^{3}i\frac{x}{{\left(1+a^{2}x^{2}\right)}^{2}}-2pBa^{3}i\frac{x}{1+a^{2}x^{2}}-s_{V}a^{2}$

**Table 3:** Parameter settings $p_{i}$ corresponding to the potentials listed in reference [47]. The latter are labelled by $\left(m_{1},m_{2}\right)$.

$\left(m_{1},m_{2}\right)$	$p_{I}$	$p_{\mathrm{II}}$	$p_{\mathrm{III}}$	$p_{\mathrm{IV}}$	$p_{V}$	*C*
(0,0)	0	0	0	0	1	$4/\sigma^{2}$
(1/2,1/2)	1	0	0	0	0	$-1/\sigma^{2}$
(1/2,0)	1	0	0	1	0	$2/\sigma^{2}$
(1/2,−1/2)	2	0	0	2	−1	$-4/\sigma^{2}$
(1,1)	0	1	0	0	0	$1/(4\sigma^{2}$)
(1,1/2)	0	1	1	0	0	$-1/(2\sigma^{2}$)
(1,0)	−1	2	2	0	0	$1/\sigma^{2}$
(1,−1/2)	−3	4	4	−1	0	$-2/\sigma^{2}$
(1,−1)	−8	8	8	−4	1	$4/\sigma^{2}$

## Data Availability

Not applicable.
